# Time course of perceptual, cognitive, physical and physiological responses to a single non‐sleep deep rest session in physically active young adults

**DOI:** 10.1111/aphw.70180

**Published:** 2026-07-01

**Authors:** Omar Boukhris, Haresh Suppiah, Manel Kerkeni, Ana Holt, Matthew Driller

**Affiliations:** ^1^ School of Allied Health, Human Services, and Sport La Trobe University Melbourne Victoria Australia

**Keywords:** autonomic function, fatigue, guided relaxation, NSDR, reaction time

## Abstract

Non‐sleep deep rest (NSDR), a brief guided relaxation technique involving slow breathing and sequential body awareness, has recently been proposed as a practical rest strategy. However, evidence on its short‐term time course and physiological responses remains limited. This study examined the effects of a brief NSDR session on perceptual, cognitive, physical, and physiological outcomes in physically active young adults. In a parallel‐group design, 102 participants (mean age: 22 ± 3 years) were randomly allocated to either a 10‐min guided NSDR protocol (n = 51) or a control condition (n = 51). Perceptual outcomes included sleepiness, fatigue, readiness to perform, as well as muscular stress, lack of activation, negative emotional state, overall stress, physical performance capacity, mental performance capacity, emotional balance and overall recovery (short recovery and stress scale), alongside cognitive (Simon task) and physical (handgrip strength and countermovement jump) performance assessed at baseline, immediately, 20 min and 40 min post‐intervention. Physiological responses (heart rate, heart rate variability and skin temperature) were recorded continuously during the intervention. Linear mixed‐effects models showed that NSDR resulted in significant Group × Time improvements across perceptual measures, including reduced sleepiness (*p* = .009, *η_p_* ^2^ = .03), fatigue (*p* = .001, *η_p_* ^2^ = .05), stress (*p* = .005, *η_p_* ^2^ = .04) and lack of activation (*p* = .011, *η_p_* ^2^ = .04), alongside enhanced mental performance capacity (*p* = .008, *η_p_* ^2^ = .04) and overall recovery (*p* = .014, *η_p_* ^2^ = .03) compared to control. Reaction time in the Simon task (Group × Time: *p* < .001, *η_p_* ^2^ = .07) was enhanced in the NSDR group immediately post‐intervention (*p* = .012), while no differences were observed at later assessments. Physiological responses showed a significant Group × Time interaction for heart rate, indicating a greater relative reduction during NSDR compared to control, alongside a smaller rise in skin temperature in the NSDR condition (*p* < .05). No significant effects were observed for physical performance outcomes (*p* > .05). These findings suggest that NSDR was associated with consistent improvements in perceptual outcomes, while cognitive findings were transient and physiological findings were modest and should be interpreted cautiously. These results support further evaluation of NSDR as a brief, time‐efficient acute rest intervention.

## INTRODUCTION

Across diverse populations, including students, professionals and athletes, sub‐optimal sleep duration and poor sleep quality are frequently reported, often due to demanding schedules, performance pressures, competition timing, travel, academic study demands and elevated stress levels (Gardani et al., 2022; Janse van Rensburg et al., 2021; Lastella et al., 2020; Walsh et al., 2021). Poor sleep compromises attention, memory, emotional regulation and work performance in broader populations (Metschura, 2023) and contributes to daytime sleepiness, which is significantly associated with poorer academic outcomes, particularly among middle and high school students (Nguyen et al., 2025). Similar consequences are evident in workplace (Pilcher & Morris, 2020) and athletic contexts (Driller et al., 2023; Halson et al., 2022), where inadequate sleep contributes to reduced performance, mood disturbances, heightened stress and slower recovery (Driller et al., 2023). As such, various rest strategies, including napping (Boukhris, Trabelsi, et al., 2024; Boukhris, Trabelsi, et al., 2025; Mesas et al., 2023), meditation (Jones et al., 2020; Li et al., 2018; Sparacio et al., 2024; Ziegler et al., 2019) and breathing exercises combined with biofeedback (Li et al., 2022), have been explored to mitigate these negative effects.

Non‐sleep deep rest (NSDR) is often described as a contemporary, secular approach that shares features with traditional Yoga Nidra practices. It is delivered via a short audio track (typically 10–20 min) involving guided relaxation through deep breathing, visualisation and body awareness exercises. Classical Yoga Nidra protocols are commonly attributed to the work of Swami Satyananda Saraswati (Bihar School of Yoga), who formalised structured practices involving rotation of awareness across body regions, breath awareness and guided imagery (Satyananda & Swami, 1998), which have since been examined and adapted in contemporary research and applied settings (Musto & Hazard Vallerand, 2023). In recent years, the term NSDR has been popularised in neuroscience and health communication (e.g. Andrew Huberman on the Huberman Lab podcast) to describe brief, accessible guided rest practices framed in physiological and attentional terms.

NSDR shares structural features with Yoga Nidra, including audio guidance, a quiet resting posture, breath cues and body‐focused attention (Musto & Hazard Vallerand, 2023). However, they differ in several important ways. Classical Yoga Nidra is embedded within a yogic and contemplative framework involving intention setting through sankalpa, structured rotation of consciousness across body regions, breath awareness that may involve counting and sessions typically lasting 20 to 40 min (Musto & Hazard Vallerand, 2023). Western adaptations such as iRest further integrate emotional inquiry and self‐reflective components (Miller, 2022; Musto & Hazard Vallerand, 2023). In contrast, NSDR employs minimal breathing cues emphasising prolonged exhalation to facilitate rapid arousal downregulation and is designed for immediate use in applied contexts without contemplative aims or repeated practice requirements (Boukhris, Suppiah, et al., 2024; Wrzeciono et al., 2024).

A previous study from our laboratory (Boukhris, Suppiah, et al., 2024) demonstrated that a brief (10 min) NSDR session significantly improved physical performance (handgrip strength), cognitive performance (reaction time and accuracy) and various perceptual measures, including emotional balance, readiness and stress levels immediately post‐intervention in 65 physically active participants, using a randomised, parallel‐group design. More recently, NSDR relaxation has also been examined in a clinical context, with a randomised controlled trial reporting reductions in depression, anxiety and perceived stress following multiple NSDR sessions in patients undergoing cardiac rehabilitation (Wrzeciono et al., 2024). However, this latter study focused on repeated‐session psychological outcomes and did not assess acute performance responses, physiological mechanisms or the short‐term persistence of NSDR effects within a single session. Consequently, it remains unclear whether the beneficial effects of NSDR persist beyond immediate testing or diminish quickly over time.

NSDR presents several practical advantages compared to napping or other relaxation strategies. Specifically, its short duration (approximately 10 min) makes it particularly suitable for athletes and individuals with busy schedules, who may find longer relaxation techniques or daytime naps challenging to incorporate regularly. Unlike napping, NSDR may avoid the sleep inertia that can follow even brief naps, which typically requires up to an hour to fully dissipate and potentially hinders subsequent performance and alertness (Mesas et al., 2023). Furthermore, NSDR requires no specialised equipment, unlike biofeedback which necessitates specific devices (Li et al., 2022), nor does it require formal training or prior experience, contrasting with techniques such as meditation and yoga that typically involve structured practice and instruction (Bucea‐Manea‐Țoniș et al., 2023; Lomas et al., 2015), making it highly accessible and easy to implement across diverse settings.

While prior work has demonstrated acute benefits of NSDR (Boukhris, Suppiah, et al., 2024), the underlying physiological processes remain poorly understood. NSDR is intended to elicit a state of deep relaxation characterised by increased parasympathetic activity, and such states have been associated with reductions in muscle tension and alterations in blood flow (Thomas & Segal, 2004). These changes provide a plausible pathway through which NSDR could transiently influence neuromuscular performance, supporting the inclusion of objective physical performance measures. More specifically, NSDR is proposed to promote deep rest without sleep primarily through modulation of autonomic nervous system activity via guided attention and controlled breathing. Slow breathing patterns with prolonged exhalation are known to reduce sympathetic activation and increase parasympathetic influence, which is commonly reflected by reductions in heart rate (HR) and changes in HR variability during relaxation and breathing‐based interventions (Balban et al., 2023; Li et al., 2018, 2022). Measures such as HR, HR variability (HRV) and skin temperature provide valuable insights into autonomic regulation during rest‐based interventions, yet these indices have not been examined during NSDR. HR and HRV, in particular, are widely recognised as robust markers of parasympathetic activity and autonomic regulation (Shaffer & Ginsberg, 2017; Stanley et al., 2013). Their inclusion may help clarify the physiological mechanisms underlying NSDR's effects and differentiate it from other rest and relaxation strategies.

Therefore, the current study aims to build upon these earlier findings by investigating the persistence of NSDR‐induced enhancements in perceptual, cognitive and physical performance at multiple time points following the intervention (immediately, 20 and 40 min post‐session) in physically active young adults. Moreover, by monitoring temperature, HR and HRV during the NSDR intervention, this study seeks to provide a deeper understanding of the physiological responses associated with NSDR practice. Clarifying both the temporal profile and physiological basis of NSDR's acute effects may establish this technique as an accessible, practical and effective rest strategy, especially in those who find it difficult to nap. We hypothesised that NSDR would improve perceptual outcomes (e.g. reduced sleepiness, fatigue and stress), enhance cognitive performance and modulate physiological markers of autonomic activity compared with a passive control condition. Given prior evidence of acute handgrip improvements following NSDR, we also hypothesised that physical performance may demonstrate short‐term enhancement. However, given mixed findings across domains, the magnitude and persistence of these effects over multiple post‐intervention time points remained to be determined.

## METHODS

### Participants

G*Power software (Version 3.1.9.2; Kiel University, Kiel, Germany) (Faul et al., 2007) was used to calculate the required sample size. The *α* level was set at .05 and statistical power at .95. Effect sizes (Cohen's *f*) were derived from partial eta‐squared values reported in our previous NSDR study (Boukhris, Suppiah, et al., 2024). The estimated effect size for reaction time in the Simon task was *f* = .32, requiring a minimum sample size of 24 participants. A total of 102 participants were recruited to account for potential drop‐out and to ensure that statistical power did not fall below 95%.

A total of 102 physically active young adults (38 females, 64 males; age 22 ± 3 years) volunteered to participate in the study. Participants were recruited from a university student population. On average, participants reported engaging in 9 ± 4 h of physical activity per week, across a variety of sports (e.g. Australian football, basketball, soccer and rugby), reflecting a physically active sample. None of the participants were classified as elite or professional athletes. According to the athlete classification framework proposed by McKay et al. (2022), participants were broadly categorised within Tier 1–2 (recreationally active to trained/developmental). All participants were free from musculoskeletal injuries and self‐reported sleep or medical disorders, and none were using medications known to affect sleep, cognition or physical performance. Written informed consent was obtained from all participants prior to participation. The study was approved by the local institutional Human Ethics Committee and conducted in accordance with the Declaration of Helsinki. Participant characteristics are summarised in Table 1. All recruited participants completed the entire study protocol and were included in the final analyses.

**TABLE 1:** Participant characteristics in the control (CON) and non‐sleep deep rest (NSDR) groups.

	CON group	NSDR group
Female participants (n)	18	20
Male participants (n)	33	31
Age (years)	21 ± 2	22 ± 3
Weight (kg)	74 ± 11	74 ± 13
Heights (cm)	174 ± 9	174 ± 11
Physical activity (hours/week)	9 ± 5	8 ± 4
Sleep duration (hours)	7 ± 1	7 ± 1
Sleep quality (score, 0–3)	2 ± 1	2 ± 1
Number of habitual nappers (>1 nap per week)	19	18
Frequency of naps per week for habitual nappers	3 ± 2	3 ± 1

*Note*: Sleep quality was assessed on a 4‐point scale (0–3), where 0 = *very good*, 1 = *fairly good*, 2 = *fairly bad* and 3 = *very bad*; higher scores indicate poorer sleep quality. Nap frequency refers only to participants classified as habitual nappers (>1 nap per week). Participants who were not habitual nappers were not included in the calculation of nap frequency.

### Experimental design

The study was designed as a multi‐outcome experimental design without a pre‐registered primary endpoint, assessing perceptual, cognitive, physical and physiological responses to a single NSDR session. On the testing day, participants were allocated to either an NSDR or a control (CON) group using a randomised, parallel‐group design, with the allocation process blinded to the researchers conducting the assessments (via participants picking up a sheet of paper with either NSDR or CON on it). To maintain blinding, research assistants supervised the NSDR and CON sessions, ensuring that the lead researchers responsible for outcome testing remained unaware of group allocation throughout data collection. Data collection was conducted across five sessions, each comprising 20 to 21 participants (total *n* = 102). Within each session, participants first completed perceptual measures together, followed by cognitive (Simon task) and physical performance assessments. Following baseline testing, participants were allocated to their respective conditions: the NSDR group remained together in a quiet, dimly lit room to complete the guided session, while the CON group was directed to a separate room for the passive control period. Post‐intervention assessments followed the same order as baseline testing.

Before the intervention, participants were instructed to report their previous night's sleep duration (‘How many hours of sleep did you obtain last night?’) and to rate their sleep quality using a single‐item measure with four response options (very good, fairly good, fairly bad and very bad).

Testing was conducted in the late morning to early afternoon. Participants in the NSDR group completed a guided 10‐min NSDR session, lying comfortably on mats in a quiet, dimly lit environment (<20 lx). The guided relaxation exercises were provided through a recorded protocol available online (https://youtu.be/AKGrmY8OSHM). Conversely, the CON group participants spent the same 10‐min period seated quietly also in a dimly lit room (<20 lx) supervised by a researcher who ensured they did not engage in relaxation activities, nap, or use electronic devices. The control condition was designed as a time‐matched passive control condition, allowing isolation of the effects of the guided NSDR protocol relative to quiet rest. An active control was not included, as the primary aim of the study was to examine the acute and short‐term effects of a brief NSDR session rather than to compare NSDR with other guided or attentional interventions. Throughout the NSDR and CON session, participants' physiological responses, including HR, HRV and skin temperature, were continuously monitored using wearable finger‐worn devices (Ultrahuman Ring Air, Ultrahuman Healthcare, Bangalore, India).

Performance measures were collected at baseline (pre‐intervention) and at three follow‐up intervals: immediately, 20 and 40 min post‐intervention. These assessments included physical performance tests (handgrip strength and countermovement jumps [CMJ] using force plates), cognitive performance evaluations (Simon task) and subjective measures including sleepiness, readiness to perform, fatigue, physical performance capacity, mental performance capacity, emotional balance, overall recovery, muscular stress, negative emotional state, lack of activation and overall stress.

**FIGURE 1:**
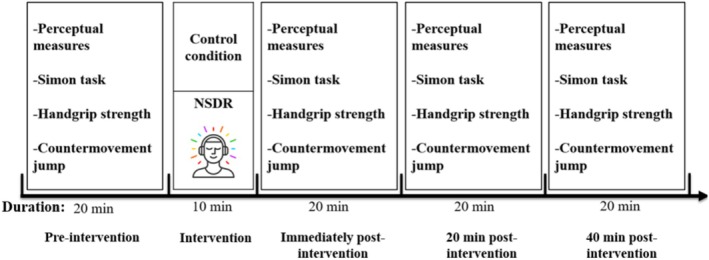
Schematic representation of the experimental design.

At the conclusion of the testing protocol, participants in the NSDR group completed a brief survey assessing their satisfaction with the intervention on a scale ranging from 0 (*very dissatisfied*) to 10 (*very satisfied*) and indicated whether they would use NSDR again (yes or no). The experimental timeline and procedure details are illustrated in Figure 1.

### NSDR protocol

The NSDR intervention consisted of a standardised 10‐min, audio‐guided session. The protocol was based on a publicly available NSDR recording (found here: https://youtu.be/AKGrmY8OSHM) narrated by Andrew Huberman, a Professor of neurobiology and ophthalmology at Stanford University, and used in prior studies (Boukhris, Suppiah, et al., 2024; Wrzeciono et al., 2024). The session incorporated guided breathing, body awareness and attentional redirection components commonly described in NSDR and Yoga Nidra‐derived practices. Participants were instructed to remain awake and breathe normally unless prompted otherwise. A research assistant was present throughout the session to monitor compliance and ensure participants followed the protocol as intended. At specific points, brief breathing cues were introduced, consisting of a deep inhalation followed by a slow, complete exhalation through the mouth with pursed lips, before returning to spontaneous breathing. The session then guided attention sequentially across the body using a moving ‘spotlight’ metaphor, beginning with the feet and lower limbs and progressing upward through the trunk, arms, face and head. During these phases, participants were instructed to notice bodily sensations and points of contact with the supporting surface, with additional breathing cues interwoven at key transitions. In the final phase, awareness was expanded to include the entire body, followed by a gradual reduction of attentional focus. The session concluded with a final deep breath and gentle reintroduction of movement before participants opened their eyes. Throughout the session, participants were instructed to direct attention towards bodily sensations and breathing, disengage from active thinking or planning and remain conscious and responsive. Participants were asked after the session whether they had fallen asleep, and none reported doing so. The full NSDR protocol script is provided in Text S1.

### Self‐reported perceptual and recovery measures

A combination of brief single‐item scales and a validated multidimensional tool (SRSS) was used to capture a broad range of perceptual and recovery‐related states. Single‐item measures of sleepiness, fatigue and readiness are widely used in the literature due to their strong face validity, sensitivity to acute changes and minimal respondent burden during repeated testing (Boukhris, Suppiah, et al., 2025; Romyn et al., 2022). The SRSS was included to provide a psychometrically robust assessment of physical, mental, emotional and overall recovery and stress. Together, these measures offer a comprehensive yet time‐efficient evaluation of perceptual responses relevant to short‐term repeated assessment of rest strategies.

Subjective sleepiness was measured using the Stanford Sleepiness Scale, which asks participants to choose from seven descriptive levels reflecting increasing drowsiness. These range from 1 (‘*Feeling active, alert, fully awake*’) to 7 (‘*Near sleep onset, unable to remain awake’*). Each descriptor corresponds to a specific sleepiness score from 1 to 7. The scale has shown strong reliability and validity, with Hoddes et al. (1973) reporting an 88% agreement in ratings.

Whole‐body fatigue was rated on a 10‐point scale adapted from Romyn et al. (2022), where 0 meant ‘*no pain or stiffness*’ and 10 indicated ‘*extreme fatigue or pain*’.

Similarly, readiness to perform was evaluated using a 10‐point scale, anchored at 0 for ‘*not at all ready*’ and 10 for ‘*fully ready*’, based on the question, ‘How ready are you to perform?’ (Romyn et al., 2022).

The SRSS is designed to evaluate an athlete's current state of recovery and stress across physical, mental, emotional and overall dimensions (Kölling et al., 2020). It is a standardised self‐report tool made up of two subscales: the Short Recovery Scale, which includes physical performance, mental performance, emotional balance and overall recovery; and the Short Stress Scale, which assesses muscular stress, low activation, negative mood and overall stress. These eight items are adapted from the broader Acute Recovery and Stress Scale and are rated using a 7‐point Likert scale ranging from 0 (‘*does not apply at all*’) to 6 (‘*fully applies*’). Internal consistency for the SRSS subscales was assessed at baseline to evaluate reliability in the present sample.

### Cognitive performance

The Simon task is a cognitive test used to assess interference control and response inhibition, two core components of cognitive control (Simon, 1990). It examines how effectively individuals handle conflicting information during response selection and execution. In this study, participants sat in front of a laptop and completed the task using a free, open‐source software programme (The Psychology Experiment Building Language ‐ PEBL, Version 2.1) (Mueller & Piper, 2014). They were instructed to respond as quickly and accurately as possible by pressing a specific key with their left or right thumb, depending on the colour of a circle (red or blue) that appeared on either side of a central fixation point. Crucially, they were told to base their response solely on the colour and ignore the position of the stimulus. A red circle required pressing the left shift key, while a blue circle required the right shift key.

The task included 70 trials presented in random order, evenly split between congruent trials, where the response key matched the stimulus location, and incongruent trials, where the response key and stimulus location were on opposite sides. Each stimulus remained on screen until the participant responded or until 1.5 s had passed, after which the next trial began automatically. Performance was evaluated using two measures: average reaction time (in milliseconds) for correct responses and accuracy (percentage of correct responses). Only correct trials were included in the reaction time analysis to reduce variability and better reflect cognitive processing speed. Accuracy was reported separately.

### Physical performance

#### Handgrip strength

Maximal grip strength of the dominant hand was measured using a Jamar Plus Digital hand dynamometer (Paterson Medical, Green Bay, WI, USA). The dynamometer was adjusted to fit each participant's hand size, and testing was performed in a standardised posture: standing upright with the shoulders adducted and neutrally rotated, elbows flexed at 90°, forearms in a neutral position, wrists extended between 0° and 30° with 0°–15° ulnar deviation, and feet placed flat on the ground. Participants completed three maximal‐effort trials separated by 30 s of passive rest, and the highest value (kg) was retained for analysis. This procedure is widely regarded as the gold standard for grip strength testing and has demonstrated excellent reliability and validity (Hamilton et al., 1994).

#### Countermovement jump

Participants performed three CMJs on a dual force platform system (ForceDecks, VALD Performance, Australia). The platforms were zeroed before each participant, after which body weight was recorded while standing still with hands placed on the hips. Following a standardised countdown (‘3–2–1’), participants were instructed to jump as high as possible using a self‐selected countermovement depth, while maintaining hands akimbo throughout the movement. Prior to the recorded trials, participants completed familiarisation jumps as part of their warm‐up to minimise learning effects. Three trials were completed with 10 s of passive rest between attempts, and the highest jump height (cm) was used for analysis. Jump height (calculated using the impulse–momentum method) was extracted using the manufacturer's software. This testing procedure has demonstrated acceptable test–retest reliability in recreational adults (Merrigan et al., 2024).

### Physiological measures

Physiological responses during the intervention were continuously monitored using a finger‐worn monitoring device (Ultrahuman Ring Air, Ultrahuman Healthcare, Bangalore, India). The device incorporates a photoplethysmography (PPG) sensor to measure HR and HRV (expressed as RMSSD), and a non‐contact temperature sensor to assess peripheral skin temperature. With the wearable devices, we recorded start and end values of HR, HRV and skin temperature during the 10‐min NSDR or CON session, as well as the average values across the full 10‐min period for HRV and temperature, and the lowest HR reached within this period.

### Statistical analysis

All outcomes were analysed in R (lme4, lmerTest, emmeans, car, effectsize). Categorical predictors were sum coded. For the four‐timepoint measures (Baseline, Post, 20‐min and 40‐min), each outcome was modelled with a Group (CON vs. NSDR) × time structure, adjusted for sex, habitual napping (yes/no), sport, sleep duration and sleep quality, with a random intercept for participant. Omnibus tests used Type III sums of squares with Satterthwaite degrees of freedom; adjusted means (EMMs) were estimated with emmeans, holding numeric covariates at their sample means and averaging over sex and nap (Kenward–Roger d.f.). When the Group × Time interaction was significant, we reported Bonferroni‐corrected simple effects (Group differences at each timepoint; Time differences within each Group), with a corrected significance threshold of *α* = .0125 for the four timepoint comparisons. For physiological measures with Start and End values (HR, HRV and temperature), defined as the first and last minute of the session, we fit analogous Group × Time2 (Start, End) mixed models. For session‐average HRV and temperature, we ran ANCOVAs with Group and the corresponding Start value as a covariate (plus the same covariates as above). Lowest HR was analysed by ANCOVA with Group and covariates. For covariates, we report estimated mean differences (ΔEMM) for categorical predictors, and regression coefficients (*β* per year, standardised *β*) for continuous predictors. Effect sizes are provided as *η_p_* ^2^ with values interpreted as follows: small (.01), medium (.06) and large (.14) (Lakens, 2013), and the proportion of variance explained was summarised using intraclass correlation coefficients (ICCs) and marginal and conditional *R* ^2^. ICCs were used to quantify the proportion of variance attributable to between‐participant differences. Marginal *R* ^2^ represents variance explained by fixed effects only, whereas conditional *R* ^2^ represents variance explained by both fixed and random effects. All predictors were specified a priori and entered simultaneously into the models; no stepwise model‐building procedures were used. Model assumptions were checked with residual diagnostics.

The significance level was set at *p* < .05. Exact *p*‐values are reported; those below .001 are denoted as *p* < .001.

## RESULTS

The characteristics of participants within the subgroup samples are summarised in Table 1.

The linear mixed‐effects models are presented in Table S1, model fit indices (ICC, marginal *R* ^2^ and conditional *R* ^2^) are reported in Table S2, and pairwise comparisons including parameter estimates, standard errors and 95% confidence intervals are reported in Tables S3 and S4. In the text, only comparisons between and within groups arising from significant main effects or interactions are reported. Descriptive values for perceptual, cognitive and physical performance are shown in Tables 2 and 3.

**TABLE 2:** Comparison of perceptual measures between non‐sleep deep rest (NSDR) and control (CON) groups at baseline, post, 20‐min and 40‐min assessments. Bold values represent significant Group × Time interactions (*p* < .05).

	CON				NSDR				Group × Time	
	Baseline	Immediately post	20 min post	40 min post	Baseline	Immediately post	20 min post	40 min post	*p* value	Effect size (*η_p_* ^2^)
Sleepiness	3.5 ± 1.2	3.8 ± 1.3	3.5 ± 1.6	3.7 ± 1.8	3.2 ± 1.1	3.2 ± 1.5	2.8 ± 1.2	2.7 ± 1.3	.**009**	.03
Fatigue	3.9 ± 0.3	4.1 ± 0.3	4.1 ± 0.3	4.4 ± 0.3	3.9 ± 0.3	3.2 ± 0.2	3.2 ± 0.2	3.3 ± 0.2	.**002**	.05
Readiness to perform	5.7 ± 1.5	5.7 ± 1.7	5.7 ± 1.7	5.5 ± 1.8	5.7 ± 1.8	6.2 ± 1.7	6.1 ± 1.7	6.0 ± 1.8	.322	.01
Physical performance capacity	3.8 ± 1.0	3.9 ± 0.9	4.0 ± 1.2	3.9 ± 1.1	3.6 ± 1.1	4.0 ± 0.9	3.9 ± 1.2	3.9 ± 1.1	.303	.01
Mental performance capacity	3.7 ± 1.0	3.8 ± 1.1	4.0 ± 1.2	3.8 ± 1.4	3.8 ± 1.1	4.4 ± 1.1	4.4 ± 1.0	4.3 ± 1.1	.**008**	.04
Emotional balance	4.3 ± 1.1	4.5 ± 1.0	4.5 ± 0.9	4.5 ± 1.1	4.3 ± 1.2	4.7 ± 1.2	4.8 ± 1.2	4.8 ± 1.1	.171	.02
Overall recovery	3.7 ± 1.2	4.1 ± 1.3	3.9 ± 1.3	3.8 ± 1.3	3.6 ± 1.2	4.1 ± 1.3	3.9 ± 1.3	3.8 ± 1.3	.**014**	.03
Muscular stress	2.7 ± 1.4	2.7 ± 1.4	2.8 ± 1.2	3.0 ± 1.2	2.8 ± 1.4	2.4 ± 1.3	2.5 ± 1.2	2.7 ± 1.2	.070	.02
Negative emotional state	1.6 ± 1.2	1.5 ± 1.1	1.7 ± 1.1	1.8 ± 1.1	1.7 ± 1.3	1.3 ± 1.0	1.4 ± 1.1	1.5 ± 1.2	.**002**	.06
Lack of activation	2.8 ± 1.1	2.8 ± 1.0	2.6 ± 1.1	2.7 ± 1.1	2.9 ± 1.3	2.3 ± 1.3	2.3 ± 1.1	2.4 ± 1.1	.**011**	.03
Overall stress	2.6 ± 1.2	2.4 ± 1.2	2.4 ± 1.2	2.5 ± 1.2	2.4 ± 1.2	1.8 ± 1.1	1.7 ± 1.1	1.9 ± 1.1	.**005**	.07

*Note*: Values are presented as raw group means ± SD. Sleepiness was assessed on a 1–7 scale, with higher scores indicating greater sleepiness. Fatigue and readiness to perform were rated on 0–10 scales, with higher scores indicating greater fatigue and greater readiness, respectively. Physical performance capacity, mental performance capacity, emotional balance and overall recovery were assessed on 0–6 scales, with higher scores indicating more favourable states. Muscular stress, negative emotional state, lack of activation and overall stress were rated on 0–6 scales, with higher scores indicating greater stress‐related symptoms. *p* values and partial eta squared (*η_p_* ^2^) correspond to the Group × Time interaction effects derived from covariate‐adjusted linear mixed‐effects models. Bonferroni correction was applied to post hoc comparisons.

**TABLE 3:** Comparison of cognitive and physical measures between non‐sleep deep rest (NSDR) and control (CON) groups at baseline, post, 20‐ and 40‐min assessments. Bold values represent significant Group × Time interactions (*p* < .05).

	CON				NSDR				Group × Time	
	Baseline	Immediately post	20 min post	40 min post	Baseline	Immediately post	20 min post	40 min post	*p* value	Effect size (*η_p_* ^2^)
Reaction time (ms)	435 ± 57	433 ± 46	411 ± 51	411 ± 48	460 ± 82	415 ± 54	401 ± 49	401 ± 39	**< .001**	.06
Accuracy during the Simon task (%)	93 ± 4	94 ± 4	93 ± 4	94 ± 4	91 ± 10	95 ± 3	95 ± 4	95 ± 4	.**021**	.03
Handgrip strength (kg)	40.7 ± 10.0	42.3 ± 11.8	41.2 ± 11.4	41.4 ± 10.9	40.1 ± 11.3	41.9 ± 12.7	42.2 ± 11.2	41.3 ± 11.2	.366	.01
Jump height (cm)	29.9 ± 7.7	30.7 ± 7.8	30.4 ± 7.9	30.3 ± 7.8	27.8 ± 7.1	29.0 ± 6.7	28.7 ± 7.1	28.6 ± 6.7	.490	.01

*Note*: Values are presented as raw group means ± SD. Reaction time (ms) was calculated from correct trials during the Simon task; lower values indicate faster responses and better performance. Accuracy during the Simon task is expressed as percentage correct, with higher values indicating better performance. Handgrip strength (kg) and jump height (cm) reflect physical performance, with higher values indicating better performance. *p* values and partial eta squared (*η_p_* ^2^) correspond to the Group × Time interaction effects derived from covariate‐adjusted linear mixed‐effects models. Bonferroni correction was applied to post hoc comparisons.

### Perceptual self‐report outcomes

Results are presented as estimated marginal means (EMMs) derived from the mixed‐effects models, which represent covariate‐adjusted model estimates. Reported ΔEMMs reflect between‐group differences in these adjusted means.

A significant Group × Time interaction was observed for sleepiness (*F*_[3,293]_ = 3.95, *p* = .009, *η_p_* ^2^ = .03, small) and fatigue (*F*_[3,293]_ = 5.53, *p* = .001, *η_p_* ^2^ = .05, small). However, no significant Group × Time interaction was observed for readiness to perform (*F*_[3,293]_ = 1.16, *p* = .322, *η_p_* ^2^ = .01, small).

**FIGURE 2:**
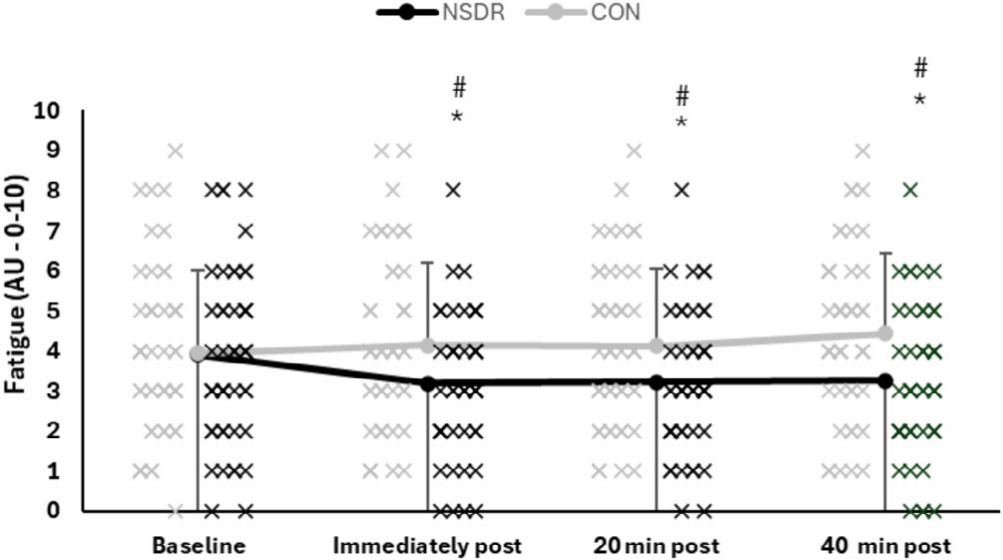
Comparison of fatigue scores between non‐sleep deep rest (NSDR) and control (CON) groups at baseline, immediately post, 20‐min and 40‐min assessments. Solid lines and error bars represent raw group means ± SD. Individual participant values are shown as ‘×’ marks. Statistical significance is based on the covariate‐adjusted linear mixed‐effects models; * indicates significant between‐group differences at the corresponding timepoint, and # indicates significant within‐group differences relative to baseline (Bonferroni corrected). Fatigue was rated on a 0–10 scale, where higher scores indicate greater fatigue.

Simple effects with Bonferroni corrections showed NSDR had significantly lower sleepiness and fatigue (Figure 2) than CON at all post‐intervention timepoints: immediately (sleepiness: ΔEMM = .68, *p* = .007; fatigue: ΔEMM = 1.03, *p* = .003), at 20 min (sleepiness: ΔEMM = .78, *p* = .003; fatigue: ΔEMM = .99, *p* = .005) and at 40 min (sleepiness: ΔEMM = 1.16, *p* < .001; fatigue: ΔEMM = 1.24, *p* < .001).

Females reported higher sleepiness (ΔEMM = .51, *p* = .030) and fatigue (ΔEMM = .97, *p* = .003), and lower readiness (ΔEMM = −.69, *p* = .028) than males, and poorer sleep quality was associated with greater sleepiness (*β* = −.65, *p* = .001) and fatigue (*β* = −1.17, *p* < .001), whereas better sleep quality was associated with greater readiness to perform (*β* = .72, *p* = .007). Higher weekly sport participation was also linked to increased fatigue (*β* = .08, *p* = .031).

### Recovery and stress outcomes

A significant Group × Time interaction was observed for mental performance capacity (*F*_[3,293]_ = 4.00, *p* = .008, *η_p_* ^2^ = .04, small), overall recovery (*F*_[3,293]_ = 3.57, *p* = .014, *η_p_* ^2^ = .03, small), negative emotional state (*F*_[3,293]_ = 4.90, *p* = .002, *η_p_* ^2^ = .05, small), lack of activation (*F*_[3,293]_ = 3.79, *p* = .011, *η_p_* ^2^ = .04, small) and overall stress (*F*_[3,293]_ = 4.44, *p* = .005, *η_p_* ^2^ = .04, small). However, no significant Group × Time interaction was observed for physical performance capacity (*F*_[3,293]_ = 1.21, *p* = .303, *η_p_* ^2^ = .01, small), emotional balance (*F*_[3,293]_ = 1.68, *p* = .171, *η_p_* ^2^ = .01, small) and muscular stress (*F*_[3,293]_ = 2.37, *p* = .070, *η_p_* ^2^ = .02, small).

Participants in the NSDR group demonstrated significantly higher mental performance capacity and significantly lower overall stress compared to CON immediately, at 20 min and at 40 min post‐intervention (mental performance capacity: immediately: ΔEMM = −.70 and *p* = .001, 20 min: ΔEMM = −.48 and *p* = .030, 40 min: ΔEMM = −.61 and *p* = .005; overall stress: ΔEMM range = .69–.71, all *p* = .001). For lack of activation, the NSDR group reported lower scores than CON at only immediately post‐intervention (ΔEMM = .58, *p* = .008). Negative emotional state was significantly lower in the NSDR group compared to CON at 20 min and 40 min post‐intervention (all ΔEMM = .45, all *p* = .041).

For the SRSS outcomes, sex predicted lower mental performance capacity (ΔEMM = −.45, *p* = .023) and higher overall stress (ΔEMM = .74, *p* < .001). Sleep quality was associated with multiple SRSS dimensions: better sleep quality predicted greater overall recovery (*β* = .70, *p* < .001), while poorer sleep quality was associated with higher muscular stress (*β* = −.54, *p* = .008), higher negative emotional state (*β* = −.36, *p* = .042), higher lack of activation (*β* = −.59, *p* < .001) and higher overall stress (*β* = −.50, *p* = .004). No significant covariate effects were found for physical performance capacity or emotional balance.

Internal consistency was acceptable for the Short Recovery Scale (*α* = .70) and moderate for the Short Stress Scale (*α* = .64).

### Cognitive performance

A significant Group × Time interaction was observed for reaction time (*F*_[3,293]_ = 6.70, *p* < .001, *η_p_* ^2^ = .07, medium) and accuracy (*F*_[3,293]_ = 3.29, *p* = .021, *η_p_* ^2^ = .03, small).

**FIGURE 3:**
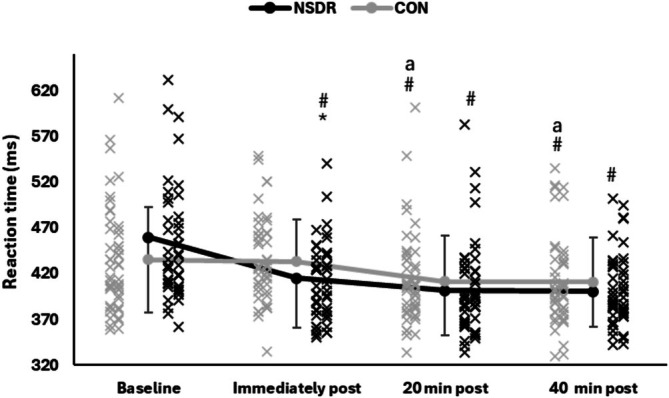
Comparison of reaction time for correct trials during the Simon task between non‐sleep deep rest (NSDR) and control (CON) groups at baseline, immediately post, 20‐min and 40‐min assessments. Solid lines and error bars represent raw group means ± SD. Individual participant values are shown as ‘×’ marks. Statistical significance is based on covariate‐adjusted linear mixed‐effects models; * indicates significant between‐group differences at the corresponding timepoint, # indicates significant within‐group differences relative to baseline, and a indicates significant within‐group differences relative to immediately post (Bonferroni corrected).

Simple effects with Bonferroni corrections showed NSDR had significantly faster reaction time than CON at immediately post‐intervention (ΔEMM = 23.6, 95% CI [5.10, 42.12], *p* = .012) (Figure 3).

Sex was a significant covariate, with females showing slower reaction times than males (ΔEMM = 18.07, *p* = .032).

In NSDR, accuracy increased from baseline at post, 20 and 40 min (all *p* < .001). CON showed no within‐group change. Between‐group differences at matched time points were not significant (all *p* > .05).

### Physical performance

No significant Group × Time interaction was observed for handgrip strength (*F*_[3,293]_ = 1.06, *p* = .366, *η_p_* ^2^ = .01, small) and CMJ (*F*_[3,293]_ = .80, *p* = .490, *η_p_* ^2^ = .008, small).

Sex was a significant covariate, with females producing lower handgrip strength (ΔEMM = −15.98, *p* < .001) and jump heights (ΔEMM = −10.54, *p* < .001) compared to males.

### Physiological measures

Descriptive values for HR, HRV and skin temperature are shown in Table 4.

**TABLE 4:** Heart rate (HR), HR variability (HRV) and skin temperature at Start and End of the 10‐min NSDR and control (CON) sessions (Mean ± SD). Bold values represent significant Group × Time interactions (*p* < .05).

	Start	CON				NSDR				Group × Time		
		End	Δ (Start to End)	Average	Lowest	Start	End	Δ (Start to End)	Average	Lowest	*p* value	Effect size (*η_p_* ^2^)
HR	87 ± 26	86 ± 21	0 ± 36	NA	68 ± 11	89 ± 24	83 ± 28	−6 ± 34	NA	66 ± 14	.**031**	.05
HRV	61 ± 41	55 ± 45	−6 ± 55	53 ± 27	NA	85 ± 54	62 ± 41	−23 ± 52	59 ± 32	NA	.172	.01
Skin temperature	29 ± 3	33 ± 3*	4 ± 2	31 ± 4	NA	30 ± 3	33 ± 3*	2 ± 2	32 ± 4	NA	.**001**	.10

* Significant difference between Start and End; NA: not applicable.

A significant Group × Time interaction was observed for HR start‐to‐end values (*F*_[1,88]_ = 4.77, *p* = .031, *η_p_* ^2^ = .05, small). The change from start to end was significantly different between groups (*p* = .031), indicating that NSDR was associated with a greater reduction in HR. However, no significant between‐group differences were observed at start (*p* = .223) or end (*p* = .066). Within‐group comparisons indicated no significant changes from start to end in either the CON (*p* = .071) or the NSDR group (*p* = .208). For lowest HR, participants in the NSDR group reached a significantly lower minimum HR compared to CON (*p* = .049).

No significant Group × Time interaction was observed for start‐to‐end HRV values (*F*_[1,94]_ = 1.89, *p* = .172, *η_p_* ^2^ = .02, small). For session‐average HRV, after adjusting for the start value, no significant between‐group differences were found (*p* = .884).

A significant Group × Time interaction was observed for start‐to‐end temperature values (*F*_[1,94]_ = 10.86, *p* = .001, *η_p_* ^2^ = .10, medium). NSDR participants showed significantly higher values at start compared to CON (*p* = .023), while no differences were observed at end (*p* = .830). Within groups, temperature increased significantly from start to end in both CON and NSDR (*p* < .001). However, the magnitude of increase was significantly greater in CON than in NSDR (*p* = .001). For session‐average temperature, after adjusting for the start value, no significant between‐group differences were found (*p* = .101).

## DISCUSSION

The present study examined perceptual, cognitive, physical and physiological outcomes following a brief NSDR intervention. The most consistent and temporally stable findings from this study were in the perceptual domain. NSDR was associated with lower sleepiness, fatigue and overall stress and higher mental performance capacity compared with CON across all post‐intervention assessments. Cognitive effects were limited to an immediate post‐intervention difference in reaction time that was not sustained over time and should be interpreted cautiously given the slightly higher baseline reaction times in the NSDR group. Physiological outcomes reflected modest differences in HR and skin temperature at the interaction level, without consistent between‐group effects at individual time points. However, physical performance was unaffected.

NSDR was associated with lower sleepiness, fatigue and overall stress across all time points, reduced lack of activation immediately post, and lower negative emotional state at 40 min compared to CON. These findings align with our previous work (Boukhris, Suppiah, et al., 2024) showing NSDR improved readiness, emotional balance, recovery and muscular stress, though sleepiness was unaffected in that study. In the present investigation, although NSDR showed lower sleepiness than CON at all post‐intervention assessments, sleepiness improved relative to baseline only after 40 min. This delayed response likely explains why our previous work (Boukhris, Suppiah, et al., 2024) that assessed only pre‐ to immediate post‐intervention did not detect a change. A possible explanation is that reductions in sleepiness require a longer period of parasympathetic dominance, whereas fatigue, stress and mood respond more rapidly to relaxation, though whether these effects are specific to NSDR or reflect general rest cannot be determined from the current design. Similar patterns have been observed in clinical populations (Wrzeciono et al., 2024) and in studies on napping (Boukhris, Trabelsi, et al., 2025; Mesas et al., 2023), meditation (Li et al., 2018) and breathing‐based relaxation (Li et al., 2022), which reduce fatigue and stress but often require longer sessions. Unlike naps, NSDR is not expected to induce sleep inertia, given that participants were instructed to remain awake throughout the session, though this was not directly measured in the present study.

A significant Group × Time interaction was observed for both reaction time and accuracy in the Simon task. Although NSDR showed faster reaction times immediately post‐intervention compared with CON, this finding should be interpreted with caution. Baseline reaction times were slightly higher in the NSDR group, and the effect appeared at one time point only. This pattern could be explained by regression to the mean or practice effects rather than an intervention effect. These findings build on our previous work (Boukhris, Suppiah, et al., 2024), which observed improvements in reaction time and accuracy immediately after NSDR using a mixed ANOVA. When additional post‐intervention time points are included and analysed using linear mixed‐effects models, only the immediate reaction time effect appears to be intervention‐specific. This may reflect a rapid reduction in mental fatigue or enhanced attentional engagement following guided relaxation. These outcomes are consistent with previous studies on mindfulness and relaxation practices, which have been shown to enhance attentional control and inhibitory processing (Datta et al., 2023; Yadav et al., 2022). In contrast, naps can also improve cognitive performance but usually require longer durations and are often followed by a period of sleep inertia that temporarily slows responses (Boukhris, Suppiah, et al., 2025; Boukhris, Trabelsi, et al., 2025; Mesas et al., 2023). These findings suggest that NSDR may offer a short‐term strategy to support attentional performance without the drawback of post‐nap sleep inertia. However, the effect was limited to the immediate post‐intervention assessment and was not sustained over time.

The effects of NSDR on physical performance were less evident than for perceptual and cognitive outcomes. No significant Group × Time interaction or group main effect was found for handgrip strength or CMJ, indicating that NSDR did not outperform CON on these measures. This contrasts with our previous work (Boukhris, Suppiah, et al., 2024), which showed handgrip improvements following NSDR. These discrepancies may reflect differences in design (immediate vs. multiple follow‐up assessments), sample size (102 vs. 65 participants, increasing variability) and participant characteristics. The absence of CMJ effects may also relate to the greater technical and coordinative demands of jumping tasks, which are less sensitive to brief rest interventions. Neuromuscular performance may require more than 10 min of relaxation to produce measurable change. Therefore, longer NSDR durations (e.g. 20 min) might be worth testing in future studies.

NSDR was associated with physiological differences compared with CON during the intervention, reflected by a greater relative reduction in HR (−6 vs. −1 bpm) and a smaller increase in skin temperature, which may be compatible with reduced sympathetic activation. However, these effects for HR were observed at the level of the Group × Time interaction, with no significant within‐group reductions or between‐group differences at individual time points, so they should be interpreted cautiously. One possible explanation for this pattern is that the final minute of the intervention coincided with the transition out of the guided session, during which participants were instructed to reorient, open their eyes and begin moving. This pattern may reflect a shift towards parasympathetic dominance, although this remains speculative and autonomic activity likely fluctuates dynamically across the session. However, HRV measures did not show significant between‐group differences, indicating that the autonomic effects of NSDR were not consistently reflected in HRV outcomes. Such findings fit with the broader literature on relaxation, breathing practices and meditation, which also report lower HR and higher HRV during periods of guided relaxation (Li et al., 2018, 2022). Our previous work (Boukhris, Suppiah, et al., 2024) proposed that NSDR may slow brainwave activity similar to light or slow‐wave sleep. This mechanism, alongside controlled breathing that reduces sympathetic tone and lowers arousal (Balban et al., 2023), may help explain the perceptual, cognitive and autonomic benefits observed here. However, the present design does not allow isolation of specific NSDR components from the general effects of quiet supine rest, and these mechanistic interpretations should therefore be considered speculative. Practically, these physiological adjustments may reflect a state that supports recovery and readiness, potentially benefiting athletes between training sessions or physically active individuals seeking to regulate stress and optimise performance in everyday contexts.

The effects of NSDR varied between participants and were associated with several baseline characteristics included as covariates in the models. Sleep quality was the strongest and most consistent covariate: those with poorer sleep reported greater sleepiness, fatigue and stress and lower recovery across both groups. While this pattern suggests that NSDR may be particularly valuable for individuals starting from a less favourable baseline, this interpretation should be treated cautiously as sleep quality was not formally tested as a moderator of the intervention effect. Sex was also associated with several outcomes, with females reporting higher sleepiness, fatigue and stress and demonstrating lower handgrip and jump performance, consistent with known sex‐related differences in neuromuscular capacity (Wilson et al., 2024) and slower reaction times (Der & Deary, 2006). These large between‐sex differences likely contributed to the absence of consistent group effects in physical performance. The absence of menstrual cycle control in female participants may have further increased variability, as strength, fatigue and recovery can fluctuate across the cycle (McNulty et al., 2020; Sims & Heather, 2018). The extent to which individual differences accounted for variance across all domains is further reflected in the model fit indices reported in Table S2. The high ICCs observed for physical outcomes (handgrip strength: .814; jump height: .920) indicate that performance on these measures was largely determined by stable between‐participant characteristics, that is, some individuals were consistently stronger or more powerful than others regardless of condition or time, leaving limited room for a brief intervention to produce detectable group‐level changes. In contrast, ICCs for perceptual and cognitive outcomes were moderate (range: .35–.78), suggesting greater within‐person fluctuation and thus more sensitivity to short‐term interventions such as NSDR. Across all outcomes, the gap between conditional *R* ^2^ and marginal *R* ^2^ was large (conditional *R* ^2^: .41–.96; marginal *R* ^2^: .05–.52), suggesting that individual differences accounted for more variance than the intervention itself. Despite this, significant Group × Time interactions were still observed for perceptual and cognitive outcomes, indicating that NSDR effects were detectable even in the presence of high between‐participant variability.

Together, NSDR was associated with benefits across some domains. However, the magnitude and detectability of its effects varied by outcome domain. Sex and sleep quality were associated with outcome levels across both groups as covariates but were not formally tested as moderators. Future studies should therefore adopt subgroup analyses and incorporate objective assessments of sleep and menstrual status to better capture the conditions under which NSDR is most effective.

Some limitations should be acknowledged. The study included multiple prespecified outcomes across perceptual, cognitive, physical and physiological domains. Although post hoc comparisons were Bonferroni‐corrected within each model, no formal adjustment was applied across outcome domains. Consequently, the number of statistical tests increases the possibility of Type I error, and findings that were transient or limited to a single assessment point should be interpreted cautiously and confirmed in future studies. Several perceptual outcomes were assessed using single‐item measures, which are practical for repeated‐measures designs but less psychometrically robust than multi‐item instruments. A further limitation concerns the internal consistency of the SRSS subscales in the present sample. Reliability was acceptable for the Short Recovery Scale (*α* = .70) and moderate for the Short Stress Scale (*α* = .64). These values are somewhat lower than those reported in German and English validation studies, where larger samples have typically yielded *α* values of .70–.74 for the Recovery scale and .76–.78 for the Stress scale (Hitzschke et al., 2015; Nässi et al., 2017), and even higher coefficients in large international validation samples (*α* = .81–.87 for Recovery; *α* = .76–.83 for Stress; Kölling et al., 2020). However, such differences are not unexpected given the smaller sample size and the brief, four‐item, state‐based nature of the SRSS, and values in the .60–.70 range remain acceptable for repeated monitoring of rapidly fluctuating constructs. Physical performance outcomes appeared to be influenced by sex, which may have increased variability and limited the detection of group‐level effects. Although individual characteristics were included as covariates rather than formally tested as moderators, future studies should be designed with sufficient power to examine whether NSDR effects differ across relevant subgroups. The control condition (i.e. quiet sitting in a dimly lit environment for 10 min) may have induced a degree of autonomic relaxation, potentially attenuating between‐group differences, particularly for physiological outcomes such as HR and HRV. More importantly, this design does not allow isolation of the specific contribution of individual NSDR components, such as guided breathing cues or attentional redirection, from the general effects of quiet rest. Future studies should incorporate both active and passive control conditions to better isolate the mechanisms underlying NSDR. Another methodological consideration concerns the postural difference between conditions. Participants in the NSDR group were supine on mats throughout the intervention, while those in the CON group remained seated. As posture independently may influence autonomic activity and subjective perceptions (e.g. HR, HRV and comfort), this difference may have contributed to observed between‐group differences beyond the specific effects of the guided NSDR protocol. This approach was adopted to minimise the likelihood of participants in the CON group falling asleep during the intervention period. Future studies should match body position across conditions to better isolate the effects of NSDR from postural influences. While the study examined short‐term effects, it did not assess whether repeated NSDR practice produces cumulative benefits. Furthermore, physiological measures were collected using a wearable ring device (Ultrahuman Ring Air) that has not yet been formally validated in peer‐reviewed publications against gold‐standard laboratory measures such as ECG for HR and HRV or standard skin thermistors for temperature. Therefore, these findings should be interpreted with considerable caution and confirmed in future studies using validated devices. Baseline psychological states and prior experience with relaxation or meditation techniques were not formally assessed. Although all participants completed a standardised familiarisation session with the NSDR protocol prior to testing and were not informed of specific study hypotheses, individual differences in prior exposure or expectations cannot be excluded, particularly for subjective outcomes. Additionally, we asked participants afterwards if they fell asleep, and no one reported doing so, though this cannot be verified without EEG recordings. Finally, the sample consisted of physically active university students, which limits generalisability to other populations such as sedentary individuals, elite athletes, who face different training and recovery demands, individuals with sleep disturbances, or clinical groups, where autonomic and perceptual responses may differ significantly.

## CONCLUSION

A single 10‐min NSDR session was associated with consistent improvements in perceptual outcomes, including reduced sleepiness, fatigue and stress. In contrast, cognitive and physiological findings were more limited and should be interpreted cautiously. Results were adjusted for individual characteristics such as sex and sleep quality, highlighting the need to consider baseline factors when applying or evaluating NSDR. Together with high enjoyment and willingness to reuse the protocol, a single NSDR session could offer a low‐cost strategy with potential benefits across different settings. While no physical performance effects were observed, NSDR may serve as a practical short‐term alternative to napping when time or circumstances limit other rest strategies. Future research should compare NSDR directly with naps and extend testing to elite athletes and clinical populations to establish contexts where it offers the greatest advantage.

## CONFLICT OF INTEREST STATEMENT

The authors declare that they have no conflicts of interest.

## ETHICS STATEMENT

The present study was approved by the Human Research Committee at La Trobe University (HEC23255).

## Supporting information


**Table S1.** Linear mixed‐effects models results for perceptual, cognitive, physical, and physiological measures.
**Table S2.** Intra‐class correlation coefficients (ICC), marginal R^2^, and conditional R^2^ for perceptual, cognitive, and physical measures.
**Table S3.** Estimated marginal means and pairwise comparisons from mixed‐effects models: between‐group contrasts (Control − NSDR) at each timepoint.
**Table S4.** Estimated marginal means and pairwise comparisons from mixed‐effects models: within‐group contrasts relative to baseline.

## Data Availability

The data that support the findings of this study are available from the corresponding author upon reasonable request.
