# Supplementary material for: Time course of perceptual, cognitive, physical and physiological responses to a single non‐sleep deep rest session in physically active young adults
Source: Appl Psychol Health Well Being. 2026 Jul 1;18(4):e70180. doi: 10.1111/aphw.70180 (PMC13321136; doi:10.1111/aphw.70180)
Supplement: Supplementary file 1 — Table S1. Linear mixed‐effects models results for perceptual, cognitive, physical, and physiological measures. Table S2. Intra‐class correlation coefficients (ICC), marginal R2, and conditional R2 for perceptual, cognitive, and physical measures. Table S3. Estimated marginal means and pairwise comparisons from mixed‐effects models: between‐group contrasts (Control − NSDR) at each timepoint. Table S4. Estimated marginal means and pairwise comparisons from mixed‐effects models: within‐group contrasts relative to baseline. [file APHW-18-0-s001.docx]

**Supplementary Text S1.** NSDR Protocol (Script)

Welcome to this 10-minute non-sleep deep rest (NSDR) protocol. Non-sleep deep rest is a powerful tool that allows you to control the relaxation state of your nervous system and your overall state of mind. It takes advantage of the fact that specific forms of breathing can place us into a state of deep relaxation by slowing our heart rate. It also uses our ability to control perception, shifting our attention away from thinking, stress, planning, or anticipation, and instead focusing on pure sensation and deep relaxation.

For this protocol, you’ll want to be seated or lying down. If you’re not already, please do so now.

Throughout the session, you’ll mostly breathe normally unless instructed otherwise. At times, you’ll be asked to inhale deeply, ideally through your nose, but through your mouth if needed. Inhale fully, then exhale completely through your mouth, pursing your lips as if through a small straw. Long, extended exhales slow the heart rate and relax the nervous system. Repeat this pattern two more times before returning to normal breathing.

Now, in your mind’s eye, imagine yourself standing over your body, holding a spotlight directed at your feet. Focus your attention on whatever your feet are in contact with socks, shoes, the floor, or even just the air. Notice any sensations: tingling, pressure, numbness. Then expand the spotlight gradually upward to include your ankles, shins, calves, thighs, hamstrings, and waist. With your lower body illuminated, take another deep inhale and exhale, repeating twice more, and imagine your lower body sinking slightly deeper into the surface beneath you.

Next, move the spotlight to your abdomen. As you inhale, feel your stomach expand outward slightly; as you exhale, feel it sink back down. Broaden your attention to include your upper abdomen, chest, and neck, and then expand further to include your arms. Bring awareness to your back, notice the points of contact with the surface beneath you, whether it’s a chair, sofa, or bed. With each exhale, imagine your back and upper body sinking another centimeter deeper into that surface.

Now move the spotlight to your face, the top of your head, and the back of your head. As you breathe normally, focus on relaxing the muscles of your face. See if you can extend your exhales slightly longer. Then expand the spotlight downward again to include your neck, chest, arms, abdomen, waist, legs, and feet. Imagine your entire body illuminated in this light. Then, in your mind’s eye, dim the spotlight, allowing the illumination to fade as your body sinks further into relaxation.

Take one more deep inhale and complete exhale, and imagine your entire body sinking into the surface beneath you. Then, gently move your toes, ankles, and knees. Shift your upper body and head slightly from side to side. Move your hands and arms gently, lifting and setting them down again. Finally, slowly open your eyes.

Notice how your nervous system, usually bombarded with sensory information, has been given a break during this protocol. You’ve directed your perceptions intentionally, choosing what sensations to attend to. This is the power of your brain: it can guide your actions and perceptions toward alertness, or as you’ve done now, into a state of deep relaxation.

**Table S1.** Linear mixed-effects models results for perceptual, cognitive, physical, and physiological measures.

|  | Fixed effect | F | P | Effect size (ηₚ²) |
| --- | --- | --- | --- | --- |
| Sleepiness | Group × Time | 3.95 | **.009** | 0.039 |
|  | Group | 10.69 | **.002** | 0.103 |
|  | Time | 2.36 | .071 | 0.024 |
|  | Sex | 4.83 | **.030** | 0.049 |
|  | Nap | 0.10 | .750 | 0.001 |
|  | Sleep duration | 1.10 | .295 | 0.012 |
|  | Sleep quality | 11.33 | **.001** | 0.109 |
|  | Physical activity | 0.05 | .820 | 0.001 |
| Fatigue | Group × Time | 5.53 | **.001** | 0.054 |
|  | Group | 8.04 | **.006** | 0.080 |
|  | Time | 2.13 | .096 | 0.021 |
|  | Sex | 9.26 | **.003** | 0.091 |
|  | Nap | 0.07 | .785 | 0.001 |
|  | Sleep duration | 1.84 | .178 | 0.019 |
|  | Sleep quality | 18.97 | **< .001** | 0.169 |
|  | Physical activity | 4.80 | **.031** | 0.049 |
| Readiness to perform | Group × Time | 1.16 | .322 | 0.012 |
|  | Group | 2.57 | .112 | 0.027 |
|  | Time | 1.48 | .219 | 0.015 |
|  | Sex | 4.98 | **.028** | 0.051 |
|  | Nap | 0.66 | .418 | 0.007 |
|  | Sleep duration | 0.10 | .752 | 0.001 |
|  | Sleep quality | 7.70 | **.007** | 0.076 |
|  | Physical activity | 0.09 | .76 | 0.001 |
| Physical performance capacity | Group × Time | 1.21 | .303 | 0.012 |
|  | Group | 0.04 | .844 | 0.000 |
|  | Time | 3.06 | **.028** | 0.030 |
|  | Sex | 2.14 | .147 | 0.023 |
|  | Nap | 0.01 | .917 | 0.000 |
|  | Sleep duration | 2.01 | .160 | 0.021 |
|  | Sleep quality | 2.79 | .098 | 0.029 |
|  | Physical activity | 1.14 | .287 | 0.012 |
| Mental performance capacity | Group × Time | 4.00 | **.008** | 0.039 |
|  | Group | 6.38 | **.013** | 0.064 |
|  | Time | 6.95 | **< .001** | 0.067 |
|  | Sex | 5.31 | **.023** | 0.054 |
|  | Nap | 0.03 | .861 | 0.000 |
|  | Sleep duration | 1.86 | .176 | 0.020 |
|  | Sleep quality | 2.96 | .088 | 0.031 |
|  | Physical activity | 0.16 | .686 | 0.002 |
| Emotional balance | Group × Time | 1.68 | .171 | 0.017 |
|  | Group | 1.17 | .281 | 0.013 |
|  | Time | 7.41 | **< .001** | 0.071 |
|  | Sex | 0.87 | .351 | 0.009 |
|  | Nap | 0.00 | .996 | 0.000 |
|  | Sleep duration | 0.26 | .612 | 0.003 |
|  | Sleep quality | 0.96 | .330 | 0.010 |
|  | Physical activity | 0.47 | .495 | 0.005 |
| Overall recovery | Group × Time | 3.57 | **.014** | 0.035 |
|  | Group | 1.42 | .236 | 0.015 |
|  | Time | 13.66 | **< .001** | 0.123 |
|  | Sex | 2.40 | .124 | 0.025 |
|  | Nap | 0.58 | .447 | 0.006 |
|  | Sleep duration | 0.51 | .474 | 0.006 |
|  | Sleep quality | 14.06 | **< .001** | 0.131 |
|  | Physical activity | 2.16 | .144 | 0.023 |
| Muscular stress | Group × Time | 2.37 | .070 | 0.024 |
|  | Group | 1.12 | .292 | 0.012 |
|  | Time | 3.68 | **.012** | 0.036 |
|  | Sex | 1.10 | .295 | 0.012 |
|  | Nap | 0.16 | .686 | 0.002 |
|  | Sleep duration | 1.02 | .315 | 0.011 |
|  | Sleep quality | 7.44 | **.008** | 0.074 |
|  | Physical activity | 2.85 | .095 | 0.030 |
| Negative emotional state | Group × Time | 4.90 | .002 | 0.048 |
|  | Group | 2.46 | .120 | 0.026 |
|  | Time | 6.79 | **< .001** | 0.065 |
|  | Sex | 3.95 | .050 | 0.048 |
|  | Nap | 0.13 | .711 | 0.001 |
|  | Sleep duration | 0.75 | .386 | 0.008 |
|  | Sleep quality | 4.06 | **.047** | 0.042 |
|  | Physical activity | 0.09 | .754 | 0.001 |
| Lack of activation | Group × Time | 3.79 | **.011** | 0.037 |
|  | Group | 3.61 | .060 | 0.037 |
|  | Time | 5.92 | **.001** | 0.057 |
|  | Sex | 2.68 | .105 | 0.028 |
|  | Nap | 0.03 | .859 | 0.000 |
|  | Sleep duration | 0.12 | .729 | 0.001 |
|  | Sleep quality | 12.72 | **.001** | 0.120 |
|  | Physical activity | 0.01 | .922 | 0.000 |
| Overall stress | Group × Time | 4.44 | **.005** | 0.044 |
|  | Group | 9.70 | **.002** | 0.095 |
|  | Time | 14.91 | **< .001** | 0.132 |
|  | Sex | 13.41 | **< .001** | 0.126 |
|  | Nap | 1.69 | .197 | 0.018 |
|  | Sleep duration | 0.06 | .805 | 0.001 |
|  | Sleep quality | 8.91 | **.004** | 0.087 |
|  | Physical activity | 0.02 | .882 | 0.000 |
| Reaction time for correct trials during Simon task | Group × Time | 6.70 | **< .001** | 0.069 |
|  | Group | 1.17 | .281 | 0.013 |
|  | Time | 35.25 | **< .001** | 0.279 |
|  | Sex | 4.74 | **.032** | 0.051 |
|  | Nap | 0.17 | .675 | 0.002 |
|  | Sleep duration | 0.99 | .322 | 0.011 |
|  | Sleep quality | 0.18 | .664 | 0.002 |
|  | Physical activity | 0.19 | .665 | 0.002 |
| Accuracy percentage during Simon task | Group × Time | 3.29 | **.021** | 0.033 |
|  | Group | 0.96 | .330 | 0.010 |
|  | Time | 8.40 | **< .001** | 0.081 |
|  | Sex | 2.24 | .138 | 0.024 |
|  | Nap | 3.49 | .065 | 0.036 |
|  | Sleep duration | 0.30 | .584 | 0.003 |
|  | Sleep quality | 0.60 | .438 | 0.007 |
|  | Physical activity | 0.88 | .350 | 0.010 |
| Handgrip strength | Group × Time | 1.06 | .366 | 0.011 |
|  | Group | 0.41 | .519 | 0.004 |
|  | Time | 4.21 | **.006** | 0.041 |
|  | Sex | 99.81 | **< .001** | 0.518 |
|  | Nap | 1.85 | .177 | 0.020 |
|  | Sleep duration | 0.09 | .763 | 0.001 |
|  | Sleep quality | 0.88 | .349 | 0.009 |
|  | Physical activity | 1.11 | .293 | 0.012 |
| Jump height | Group × Time | 0.80 | .490 | 0.008 |
|  | Group | 0.39 | .534 | 0.004 |
|  | Time | 8.49 | **< .001** | 0.082 |
|  | Sex | 99.96 | **< .001** | 0.524 |
|  | Nap | 3.06 | .083 | 0.033 |
|  | Sleep duration | 0.56 | .455 | 0.006 |
|  | Sleep quality | 0.87 | .352 | 0.010 |
|  | Physical activity | 1.90 | .171 | 0.020 |
| Start – End heart rate | Group × Time | 4.77 | **.031** | 0.051 |
|  | Group | 0.19 | .662 | 0.002 |
|  | Time | 0.15 | .695 | 0.001 |
|  | Sex | 0.55 | .458 | 0.006 |
|  | Nap | 0.09 | .763 | 0.001 |
|  | Sleep duration | 3.01 | **.048** | 0.046 |
|  | Sleep quality | 0.76 | .385 | 0.009 |
|  | Physical activity | 0.19 | .661 | 0.002 |
| Lowest heart rate | Group | 3.98 | **.049** | 0.050 |
|  | Sex | 3.91 | .051 | 0.050 |
|  | Nap | 0.02 | .886 | 0.0002 |
|  | Sleep duration | 2.51 | .116 | 0.030 |
|  | Sleep quality | 1.46 | .230 | 0.020 |
|  | Physical activity | 2.02 | .158 | 0.020 |
| Start – End heart rate variability | Group × Time | 1.89 | .172 | 0.020 |
|  | Group | 4.82 | **.030** | 0.051 |
|  | Time | 7.60 | **.006** | 0.075 |
|  | Sex | 0.39 | .533 | 0.004 |
|  | Nap | 1.98 | .161 | 0.022 |
|  | Sleep duration | 3.08 | .082 | 0.034 |
|  | Sleep quality | 0.0007 | .978 | 0.000 |
|  | Physical activity | 3.17 | .078 | 0.034 |
| Average heart rate variability | Group | 0.02 | .884 | 0.0002 |
|  | HRV start | 26.23 | **< .001** | 0.230 |
|  | Sex | 0.04 | .825 | 0.001 |
|  | Nap | 1.14 | .287 | 0.010 |
|  | Sleep duration | 0.67 | .412 | 0.007 |
|  | Sleep quality | 0.20 | .650 | 0.002 |
|  | Physical activity | 3.02 | .085 | 0.030 |
| Start – End temperature | Group × Time | 10.86 | **.001** | 0.104 |
|  | Group | 1.75 | .189 | 0.019 |
|  | Time | 235.14 | **< .001** | 0.714 |
|  | Sex | 3.01 | .086 | 0.033 |
|  | Nap | 5.43 | **.021** | 0.058 |
|  | Sleep duration | 0.56 | .452 | 0.006 |
|  | Sleep quality | 1.31 | .254 | 0.015 |
|  | Physical activity | 0.13 | .714 | 0.001 |
| Average temperature | Group | 2.73 | .101 | 0.030 |
|  | Temperature Start | 385.50 | **< .001** | 0.814 |
|  | Sex | 3.02 | .085 | 0.033 |
|  | Nap | 1.95 | .166 | 0.022 |
|  | Sleep duration | 0.21 | .645 | 0.002 |
|  | Sleep quality | 0.73 | .393 | 0.008 |
|  | Physical activity | 0.24 | .623 | 0.002 |

Note. Results are presented from linear mixed-effects models. The Group × Time interaction represents the primary test of the intervention effect. Significant effects (p < 0.05) are shown in bold. Main effects and covariates are provided for completeness.

**Table S2.** Intra-class correlation coefficients (ICC), marginal R², and conditional R² for perceptual, cognitive, and physical measures.

|  | Adjusted ICC | Conditional R^2^ | Marginal R^2^ |
| --- | --- | --- | --- |
| Sleepiness | 0.537 | 0.611 | 0.160 |
| Fatigue | 0.697 | 0.769 | 0.239 |
| Readiness to perform | 0.654 | 0.694 | 0.115 |
| Physical performance capacity | 0.651 | 0.685 | 0.096 |
| Mental performance capacity | 0.611 | 0.658 | 0.121 |
| Emotional balance | 0.775 | 0.787 | 0.050 |
| Overall recovery | 0.741 | 0.792 | 0.197 |
| Muscular stress | 0.777 | 0.809 | 0.140 |
| Negative emotional state | 0.830 | 0.849 | 0.115 |
| Lack of activation | 0.678 | 0.730 | 0.162 |
| Overall stress | 0.783 | 0.836 | 0.246 |
| Mean reaction time during Simon task | 0.632 | 0.687 | 0.151 |
| Accuracy during Simon task | 0.350 | 0.412 | 0.095 |
| Handgrip strength | 0.814 | 0.903 | 0.479 |
| Jump height | 0.920 | 0.962 | 0.520 |

Note. ICC represents the proportion of variance attributable to between-participant differences. Marginal R² reflects variance explained by fixed effects, and conditional R² reflects variance explained by both fixed and random effects.

**Table S3.** Estimated marginal means and pairwise comparisons from mixed-effects models: between-group contrasts (Control − NSDR) at each timepoint.

| Outcome | Timepoint | Estimate | SE | 95% CI | p |
| --- | --- | --- | --- | --- | --- |
| Sleepiness | Pre | 0.265 | 0.274 | [-0.275, 0.804] | .335 |
|  | Post | 0.685 | 0.274 | [0.145, 1.224] | **.013** |
|  | 20 min | 0.785 | 0.274 | [0.245, 1.324] | **.005** |
|  | 40 min | 1.165 | 0.274 | [0.624, 1.706] | **< .001** |
| Fatigue | Pre | 0.337 | 0.361 | [-0.376, 1.050] | .352 |
|  | Post | 1.156 | 0.361 | [0.443, 1.870] | **.002** |
|  | 20 min | 1.094 | 0.361 | [0.382, 1.810] | **.003** |
|  | 40 min | 1.336 | 0.361 | [0.622, 2.050] | **< .001** |
| Readiness to perform | Pre | -0.177 | 0.346 | [-0.861, 0.506] | .609 |
|  | Post | -0.657 | 0.346 | [-1.341, 0.026] | .059 |
|  | 20 min | -0.457 | 0.346 | [-1.141, 0.226] | .188 |
|  | 40 min | -0.624 | 0.347 | [-1.309, 0.062] | .074 |
| Physical performance capacity | Pre | 0.114 | 0.202 | [-0.284, 0.512] | .571 |
|  | Post | -0.166 | 0.202 | [-0.564, 0.232] | .412 |
|  | 20 min | 0.034 | 0.202 | [-0.364, 0.432] | .865 |
|  | 40 min | -0.12 | 0.202 | [-0.519, 0.279] | .553 |
| Mental performance capacity | Pre | -0.082 | 0.221 | [-0.518, 0.354] | .712 |
|  | Post | -0.702 | 0.221 | [-1.138, -0.266] | **.002** |
|  | 20 min | -0.482 | 0.221 | [-0.918, -0.046] | **.031** |
|  | 40 min | -0.617 | 0.221 | [-1.053, -0.180] | **.006** |
| Emotional balance | Pre | -0.022 | 0.219 | [-0.455, 0.411] | .92 |
|  | Post | -0.242 | 0.219 | [-0.675, 0.191] | .271 |
|  | 20 min | -0.322 | 0.219 | [-0.755, 0.111] | .143 |
|  | 40 min | -0.281 | 0.219 | [-0.714, 0.153] | .202 |
| Overall recovery | Pre | 0.059 | 0.238 | [-0.411, 0.530] | .804 |
|  | Post | -0.241 | 0.238 | [-0.711, 0.230] | .314 |
|  | 20 min | -0.381 | 0.238 | [-0.851, 0.090] | .112 |
|  | 40 min | -0.459 | 0.238 | [-0.930, 0.012] | .056 |
| Muscular stress | Pre | -0.028 | 0.248 | [-0.519, 0.463] | .911 |
|  | Post | 0.352 | 0.248 | [-0.139, 0.843] | .158 |
|  | 20 min | 0.312 | 0.248 | [-0.179, 0.803] | .211 |
|  | 40 min | 0.326 | 0.249 | [-0.166, 0.817] | .192 |
| Negative emotional state | Pre | 0.30 | 0.218 | [-0.401, 0.463] | .888 |
|  | Post | 0.350 | 0.218 | [-0.081, 0.783] | .11 |
|  | 20 min | 0.450 | 0.218 | [0.018, 0.883] | **.04** |
|  | 40 min | 0.450 | 0.219 | [0.017, 0.883] | **.04** |
| Lack of activation | Pre | 0.020 | 0.217 | [-0.408, 0.449] | .92 |
|  | Post | 0.580 | 0.217 | [0.151, 1.009] | **.008** |
|  | 20 min | 0.420 | 0.217 | [-0.008, 0.849] | .054 |
|  | 40 min | 0.419 | 0.218 | [-0.010, 0.849] | .055 |
| Overall stress | Pre | 0.294 | 0.212 | [-0.125, 0.712] | .167 |
|  | Post | 0.714 | 0.212 | [0.295, 1.132] | **.001** |
|  | 20 min | 0.714 | 0.212 | [0.295, 1.132] | **.001** |
|  | 40 min | 0.694 | 0.212 | [0.275, 1.113] | **.001** |
| Mean reaction time during Simon task | Pre | -11.7 | 9.33 | [-30.07, 6.77] | .213 |
|  | Post | 23.6 | 9.37 | [5.10, 42.12] | **.012** |
|  | 20 min | 12.3 | 9.33 | [-6.16, 30.68] | .190 |
|  | 40 min | 10.2 | 9.35 | [-8.25, 28.68] | .276 |
| Accuracy during Simon task | Pre | 1.54 | 1.03 | [-0.494, 3.565] | .137 |
|  | Post | -1.50 | 1.04 | [-3.543, 0.549] | .150 |
|  | 20 min | -1.57 | 1.03 | [-3.595, 0.462] | .129 |
|  | 40 min | -1.37 | 1.03 | [-3.409, 0.665] | .185 |
| Handgrip strength | Pre | -0.376 | 1.66 | [-3.66, 2.91] | .820 |
|  | Post | -0.571 | 1.66 | [-3.86, 2.71] | .731 |
|  | 20 min | -2.019 | 1.66 | [-5.30, 1.26] | .225 |
|  | 40 min | -1.021 | 1.66 | [-4.31, 2.27] | .539 |
| Jump height | Pre | 0.995 | 1.05 | [-1.09, 3.08] | .345 |
|  | Post | 0.542 | 1.05 | [-1.54, 2.63] | .607 |
|  | 20 min | 0.646 | 1.05 | [-1.44, 2.73] | .540 |
|  | 40 min | 0.363 | 1.05 | [-1.72, 2.45] | .730 |

Note. Estimates represent mean differences between Control and NSDR groups derived from covariate-adjusted linear mixed-effects models. Positive estimates indicate higher values in the Control group relative to NSDR; negative estimates indicate higher values in the NSDR group. 95% CI = 95% confidence interval. SE = standard error. Bonferroni correction was applied to post hoc comparisons. Bold values indicate significant between-group differences (p < 0.05).

**Table S4.** Estimated marginal means and pairwise comparisons from mixed-effects models: within-group contrasts relative to baseline.

| Outcome | Group | Contrast | Estimate | SE | 95% CI | p |
| --- | --- | --- | --- | --- | --- | --- |
| Sleepiness | CON | Pre − Post | -0.34 | 0.185 | [-0.832, 0.152] | .406 |
|  |  | Pre − 20min | -0.06 | 0.185 | [-0.552, 0.432] | 1 |
|  |  | Pre − 40min | -0.34 | 0.185 | [-0.832, 0.152] | .406 |
|  | NSDR | Pre − Post | 0.08 | 0.185 | [-0.412, 0.572] | 1 |
|  |  | Pre − 20min | 0.46 | 0.185 | [-0.032, 0.952] | .082 |
|  |  | Pre − 40min | 0.56 | 0.187 | [0.065, 1.056] | **.017** |
| Fatigue | CON | Pre − Post | -0.125 | 0.197 | [-0.647, 0.397] | 1 |
|  |  | Pre − 20min | -0.125 | 0.197 | [-0.647, 0.397] | 1 |
|  |  | Pre − 40min | -0.479 | 0.197 | [-1.000, 0.043] | .092 |
|  | NSDR | Pre − Post | 0.694 | 0.195 | [0.177, 1.211] | **.003** |
|  |  | Pre − 20min | 0.633 | 0.195 | [0.116, 1.150] | **.008** |
|  |  | Pre − 40min | 0.52 | 0.196 | [0.000, 1.041] | **.05** |
| Readiness to perform | CON | Pre − Post | 0.02 | 0.203 | [-0.518, 0.558] | 1 |
|  |  | Pre − 20min | -0.02 | 0.203 | [-0.558, 0.518] | 1 |
|  |  | Pre − 40min | 0.26 | 0.203 | [-0.278, 0.798] | 1 |
|  | NSDR | Pre − Post | -0.46 | 0.203 | [-0.998, 0.078] | .143 |
|  |  | Pre − 20min | -0.3 | 0.203 | [-0.838, 0.238] | .839 |
|  |  | Pre − 40min | -0.186 | 0.204 | [-0.728, 0.356] | 1 |
| Physical performance capacity | CON | Pre − Post | -0.08 | 0.119 | [-0.395, 0.235] | 1 |
|  |  | Pre − 20min | -0.18 | 0.119 | [-0.495, 0.135] | .779 |
|  |  | Pre − 40min | -0.02 | 0.119 | [-0.335, 0.295] | 1 |
|  | NSDR | Pre − Post | -0.36 | 0.119 | [-0.675, -0.045] | **.016** |
|  |  | Pre − 20min | -0.26 | 0.119 | [-0.575, 0.055] | .174 |
|  |  | Pre − 40min | -0.254 | 0.119 | [-0.571, 0.063] | .203 |
| Mental performance capacity | CON | Pre − Post | -0.02 | 0.137 | [-0.384, 0.344] | 1 |
|  |  | Pre − 20min | -0.22 | 0.137 | [-0.584, 0.144] | .659 |
|  |  | Pre − 40min | 0.04 | 0.137 | [-0.324, 0.404] | 1 |
|  | NSDR | Pre − Post | -0.64 | 0.137 | [-1.004, -0.276] | **< .001** |
|  |  | Pre − 20min | -0.62 | 0.137 | [-0.984, -0.256] | **< .001** |
|  |  | Pre − 40min | -0.495 | 0.138 | [-0.862, -0.128] | **.002** |
| Emotional balance | CON | Pre − Post | -0.16 | 0.103 | [-0.434, 0.114] | .732 |
|  |  | Pre − 20min | -0.16 | 0.103 | [-0.434, 0.114] | .732 |
|  |  | Pre − 40min | -0.12 | 0.103 | [-0.394, 0.154] | 1 |
|  | NSDR | Pre − Post | -0.38 | 0.103 | [-0.654, -0.106] | **.002** |
|  |  | Pre − 20min | -0.46 | 0.103 | [-0.734, -0.186] | **< .001** |
|  |  | Pre − 40min | -0.379 | 0.104 | [-0.654, -0.103] | **.002** |
| Overall recovery | CON | Pre − Post | -0.36 | 0.121 | [-0.680, -0.040] | **.018** |
|  |  | Pre − 20min | -0.2 | 0.121 | [-0.520, 0.120] | .589 |
|  |  | Pre − 40min | -0.08 | 0.121 | [-0.400, 0.240] | 1 |
|  | NSDR | Pre − Post | -0.66 | 0.121 | [-0.980, -0.340] | **< .001** |
|  |  | Pre − 20min | -0.64 | 0.121 | [-0.960, -0.320] | **< .001** |
|  |  | Pre − 40min | -0.598 | 0.121 | [-0.921, -0.276] | **< .001** |
| Muscular stress | CON | Pre − Post | -0.04 | 0.116 | [-0.349, 0.269] | 1 |
|  |  | Pre − 20min | -0.14 | 0.116 | [-0.449, 0.169] | 1 |
|  |  | Pre − 40min | -0.3 | 0.116 | [-0.609, 0.009] | .063 |
|  | NSDR | Pre − Post | 0.34 | 0.116 | [0.031, 0.649] | **.023** |
|  |  | Pre − 20min | 0.2 | 0.116 | [-0.109, 0.509] | .522 |
|  |  | Pre − 40min | 0.054 | 0.117 | [-0.258, 0.365] | 1 |
| Negative emotional state | CON | Pre − Post | 0.1 | 0.09 | [-0.138, 0.338] | 1 |
|  |  | Pre − 20min | -0.02 | 0.09 | [-0.258, 0.218] | 1 |
|  |  | Pre − 40min | -0.14 | 0.09 | [-0.378, 0.098] | .718 |
|  | NSDR | Pre − Post | 0.42 | 0.09 | [0.182, 0.658] | **< .001** |
|  |  | Pre − 20min | 0.4 | 0.09 | [0.162, 0.638] | **< .001** |
|  |  | Pre − 40min | 0.279 | 0.09 | [0.039, 0.519] | **.013** |
| Lack of activation | CON | Pre − Post | 0 | 0.123 | [-0.326, 0.326] | 1 |
|  |  | Pre − 20min | 0.14 | 0.123 | [-0.186, 0.466] | 1 |
|  |  | Pre − 40min | 0.04 | 0.123 | [-0.286, 0.366] | 1 |
|  | NSDR | Pre − Post | 0.56 | 0.123 | [0.234, 0.886] | **< .001** |
|  |  | Pre − 20min | 0.54 | 0.123 | [0.214, 0.866] | **< .001** |
|  |  | Pre − 40min | 0.439 | 0.123 | [0.111, 0.766] | **.003** |
| Overall stress | CON | Pre − Post | 0.18 | 0.098 | [-0.081, 0.441] | .406 |
|  |  | Pre − 20min | 0.2 | 0.098 | [-0.061, 0.461] | .254 |
|  |  | Pre − 40min | 0.1 | 0.098 | [-0.161, 0.361] | 1 |
|  | NSDR | Pre − Post | 0.6 | 0.098 | [0.339, 0.861] | **< .001** |
|  |  | Pre − 20min | 0.62 | 0.098 | [0.359, 0.881] | **< .001** |
|  |  | Pre − 40min | 0.5 | 0.099 | [0.238, 0.763] | **< .001** |
| Reaction time | CON | Pre − Post | 0.201 | 5.71 | [-14.980, 15.400] | 1 |
|  |  | Pre − 20min | 24.263 | 5.63 | [9.300, 39.200] | **< .001** |
|  |  | Pre − 40min | 23.24 | 5.67 | [8.160, 38.300] | **< .001** |
|  | NSDR | Pre − Post | 35.461 | 5.69 | [20.330, 50.600] | **< .001** |
|  |  | Pre − 20min | 48.176 | 5.7 | [33.040, 63.300] | **< .001** |
|  |  | Pre − 40min | 45.11 | 5.7 | [29.970, 60.200] | **< .001** |
| Accuracy | CON | Pre − Post | -1.134 | 0.845 | [-3.380, 1.110] | 1 |
|  |  | Pre − 20min | -0.555 | 0.834 | [-2.770, 1.660] | 1 |
|  |  | Pre − 40min | -0.909 | 0.84 | [-3.140, 1.320] | 1 |
|  | NSDR | Pre − Post | -4.166 | 0.825 | [-6.360, -1.970] | **< .001** |
|  |  | Pre − 20min | -3.657 | 0.826 | [-5.850, -1.460] | **< .001** |
|  |  | Pre − 40min | -3.816 | 0.826 | [-6.010, -1.620] | **< .001** |
| Handgrip strength | CON | Pre − Post | -1.629 | 0.712 | [-3.520, 0.262] | .137 |
|  |  | Pre − 20min | -0.451 | 0.712 | [-2.342, 1.440] | 1 |
|  |  | Pre − 40min | -0.663 | 0.712 | [-2.554, 1.228] | 1 |
|  | NSDR | Pre − Post | -1.824 | 0.712 | [-3.715, 0.067] | .065 |
|  |  | Pre − 20min | -2.094 | 0.712 | [-3.985, -0.203] | **.021** |
|  |  | Pre − 40min | -1.308 | 0.717 | [-3.212, 0.596] | .414 |
| Jump height | CON | Pre − Post | -0.782 | 0.292 | [-1.558, -0.006] | **.047** |
|  |  | Pre − 20min | -0.494 | 0.292 | [-1.270, 0.282] | .552 |
|  |  | Pre − 40min | -0.438 | 0.292 | [-1.214, 0.338] | .81 |
|  | NSDR | Pre − Post | -1.235 | 0.298 | [-2.028, -0.443] | **< .001** |
|  |  | Pre − 20min | -0.844 | 0.298 | [-1.636, -0.052] | **.03** |
|  |  | Pre − 40min | -1.07 | 0.3 | [-1.868, -0.273] | **.003** |

Note. Estimates represent within-group mean differences relative to baseline derived from covariate-adjusted linear mixed-effects models. Positive estimates indicate a reduction from baseline; negative estimates indicate an increase from baseline. 95% CI = 95% confidence interval. SE = standard error. Bonferroni correction was applied to post hoc comparisons. Bold values indicate significant differences (p < 0.05).
